# *In-situ* Functionalization of Metal Electrodes for Advanced Asymmetric Supercapacitors

**DOI:** 10.3389/fchem.2019.00512

**Published:** 2019-07-16

**Authors:** Leimeng Sun, Xinghui Wang, Yurong Wang, Dongyang Xiao, Weifan Cai, Yuan Jing, Yanrong Wang, Fangjing Hu, Qing Zhang

**Affiliations:** ^1^MOE Key Laboratory of Fundamental Physical Quantities Measurement & Hubei Key Laboratory of Gravitation and Quantum Physics, PGMF and School of Physics, Huazhong University of Science and Technology, Wuhan, China; ^2^College of Physics and Information Engineering, Institute of Micro-Nano Devices and Solar Cells, Fuzhou University, Fuzhou, China; ^3^NOVITAS, Nanoelectronics Centre of Excellence, School of Electrical and Electronic Engineering, Nanyang Technological University, Singapore, Singapore

**Keywords:** asymmetric supercapacitors, carbon nanotubes, carbon cloth, *in-situ* functionalization, metal electrode

## Abstract

Nanostructured metal-based compound electrodes with excellent electrochemical activity and electrical conductivity are promising for high-performance energy storage applications. In this paper, we report an asymmetric supercapacitor based on Ti and Cu coated vertical-aligned carbon nanotube electrodes on carbon cloth. The active material is achieved by *in-situ* functionalization using a high-temperature annealing process. Scanning and transmission electron microscopy and Raman spectroscopy confirm the detailed nanostructures and composition of the electrodes. The TiC@VCC and Cu_x_S@VCC electrodes show a high specific capacity of 200.89 F g^−1^ and 228.37 F g^−1^, respectively, and good capacitive characteristics at different scan speeds. The excellent performance can be attributed to a large surface area to volume ratio and high electrical conductivity of the electrodes. Furthermore, an asymmetric supercapacitor is assembled with TiC@VCC as anode and Cu_x_S@VCC as cathode. The full device can operate within the 0–1.4 V range, and shows a maximum energy density of 9.12 Wh kg^−1^ at a power density of 46.88 W kg^−1^. These findings suggest that the metal-based asymmetric electrodes have a great potential for supercapacitor applications.

## Introduction

Supercapacitors (SCs) with outstanding power densities and cycling performances have become one of the most promising power sources for next generation microelectronics and portable electronic products (Wang, 2010). However, their low energy density is one of major barriers for commercialization and practical applications of SCs. For example, the SCs based on carbon materials could hardly satisfy the energy demands for most practical applications in comparison with metal ion batteries. According to the energy (*E*) and capacitance (*C*) relationship, i.e., $E=\frac{1}{2}CV^{2}$, where *V* stands for the working voltage of SC, asymmetric supercapacitors (ASCs) based on novel materials are considered to be capable of improving the energy densities from two aspects. One is that the operation potential window is effectively broadened through the asymmetric design. The other one is that, by applying functional nanomaterials to electrodes to introduce the faradic capacitance, the specific capacitance of the electrodes can be significantly enhanced. As a result, ASCs based on nanostructured metal oxides have shown a significant improvement in energy density, and an operating potential of 2.0 V for ASCs have been achieved (Xiao et al., 2012). However, the metal oxides based ASCs suffer from poor conductivities and low power densities (Zhu et al., 2015). In order to solve these problems, new electroactive materials apart from transition metal oxides need to be investigated, to optimize the performance of electrodes in ASCs.

Transition metal oxides were considered as promising candidates for new energy storage material due to the introduction of pseudo capacitance that was able to significantly increase specific capacitance when compared with traditional carbon based electrodes. However, transition metal oxide are usually of poor conductivity and could not deliver high current and power densities. Transition metal carbides exhibit both fascinating energy storage performances and outstanding conductivities, when compared with transition metal oxides. Two-dimensional metal carbides have demonstrated ultrahigh specific volumetric capacitances (Lukatskaya et al., 2013). In addition, compared with metal oxides, transition metal carbides usually have better cycling performances. For example, supercapacitor based on a tubular TiC fiber nanostructured electrode was fabricated and tested for more than 150,000 cycles at a high temperature of 65°C (Xia et al., 2015). Moreover, layered titanium carbide Ti_3_C_2_ has also been proved to be a promising negative electrode material with a high mass loading of 7.6 mg cm^−2^ and a high specific capacitance of 112 F g^−1^ in a stable potential window (−0.9 to −0.3 V refer to Ag/AgCl electrode) (Lin and Zhang, 2015). Transition metal sulfides (e.g., MoS_2_, NiCo_2_S_4_, Ni_3_S_2_) based electrodes have also been extensively studied with ever improving intrinsic conductivity (Acerce et al., 2015; Fu et al., 2015; Li et al., 2015). Copper sulfides (Cu_x_S) have been used in electrochemical devices for gas sensing application (Sagade and Sharma, 2008) and lithium-ion batteries (Chung and Sohn, 2002), owing to their superior conductivity and great specific capacitance. Furthermore, acting as positive electrodes of supercapacitors, Cu_x_S presents a metal-like conductivity of ~1 × 10^3^ S cm^−1^ (Mazor et al., 2009) and remarkable specific capacitance of 110 F g^−1^ (Zhu et al., 2012). To better evaluate the capacitance of CuS based electrodes, an asymmetric supercapacitor cell, constructed with nanostructured CuS networks as the cathode and activated carbon as anode, was demonstrated with a high specific capacity of 49.8 mAh g^−1^ at a current density of 1 A g^−1^, and the maximum energy density is 17.7 Wh kg^−1^ at a power density of 504 W kg^−1^ (Fu et al., 2016).

The structures of electrodes are also important for optimizing the performance of supercapacitors. Recently, three-dimensional nanocarbon electrodes made from carbon nanotubes (CNTs) on carbon cloth (CC) were applied to electrochemical cells. A nickel-zinc battery based on a 3D hierarchical carbon nanofiber-CC electrode was reported to have a power density of 6.09 mWh cm^−3^ and an energy density of 355.7 Wh kg^−1^ (Liu et al., 2016). A lithium-ion battery with 3D carbon nanostructures as its electrodes can be consistently operated for more than 8,000 cycles (Wang et al., 2015). Furthermore, low-dimensional metal-organic frameworks (LD MOFs) have attracted increasing attention in recent years, which successfully combine the unique properties of MOFs, with the distinctive physical and chemical properties of LD nanomaterials (Xu et al., 2017, 2018; Liu et al., 2019).

Herein, we report on an asymmetric supercapacitor constructed with TiC and Cu_x_S as its anode and cathode, respectively. TiC@VCC and CuxS@VCC are chosen as negative and positive electrode, respectively, due to the potential window of the materials. To optimize the electrochemical performance of the device, we have incorporated a vertical-aligned carbon nanotube (VACNT) array on carbon cloth as the electrodes. Different from other reported CNT arrays, the VACNT array employed here is of low density and superior conductivity, and has demonstrated as promising electrochemical electrodes (Sun et al., 2015, 2016, 2017). The array with well-distributed VACNTs on carbon cloth (VCC) forms a 3D nanostructure with a large surface to volume ratio and ultra-straight morphology (Wang et al., 2016). A high-temperature annealing process is conducted to *in-situ* functionalize the metal-coated VCC electrodes for the anode and cathode. A large specific capacitance of 200.89 F g^−1^ in a potential window of −0.7–0.1 V and 228.37 F g^−1^ in −0.1–0.7 V are obtained. Moreover, a full device based on these electrodes shows a high energy density of 9.12 Wh kg^−1^ and power density of 46.88 W kg^−1^. Our findings suggest a feasible approach to achieve SCs with both high energy densities and high power densities.

## Experimental

### Preparation of VACNT Array on Carbon Cloth

Well-distributed VACNTs were grown on a piece of flexible carbon cloth as current collectors. Firstly, an ultrathin Ni/Al_2_O_3_ bi-layer catalyst was deposited through a plasma-enhanced CVD (PECVD) system (Wang et al., 2014a) and the CNT grew in a mixture of ammonia/acetylene (240/60 sccm) gas atmosphere under 120 W plasma at 800°C.

### Preparation of TiC@VCC and Cu_x_S@VCC Electrodes

After the fabrication of VCC, 500 nm titanium and copper were coated on the VACNT array using a Denton RF/DC magnetron sputtering system in which a Ti target (99.999%) and a Cu target (99.99%) were sputtered at a current of 0.7 A and 0.4 A under a pure Argon atmosphere, respectively. *In-situ* functionalization processes for both electrodes were conducted in a thermal chemical vapor deposition (TCVD) furnace. The sulfur powder was placed in an Al_2_O_3_ ceramic boat and heated in a low temperature zone and the two metal-coated VCC electrodes were placed in a high temperature zone of 700°C for about 30 min in a mixed gas (Ar/H_2_) atmosphere.

### Materials Characterization and Electrochemical Measurement

The structure and morphology of the electrodes were characterized with a SEM system (LEO 1550 Gemini) and TEM (JEM 2100 FJEOL), respectively. A Raman system (WITec) with a 532 nm wavelength excitation was applied for Raman spectrum measurements. The mass loading of these as-grown TiC or Cu_x_S nanocomposite were 0.12 and 0.65 mg/cm^2^, respectively, which were determined by the difference before and after material deposition and functionalization with an analytical balance (Mettler Toledo XP 26, 0.002 mg).

Electrochemical measurements of the electrode were carried out through an electrochemical workstation (Autolab/M101) in a 1.0 M LiCl aqueous electrolyte under a three-electrode measurement setup with a standard Ag/AgCl reference electrode. For full device test, a standard CR-2032 coin cell testing system was built and tested as a whole, in which TiC@VCC and Cu_x_S@VCC electrodes were used as the anode and cathode, respectively. A membrane was used as the separator and 1.0 M LiCl as the aqueous electrolyte.

## Results and Discussion

**Figure 1:**
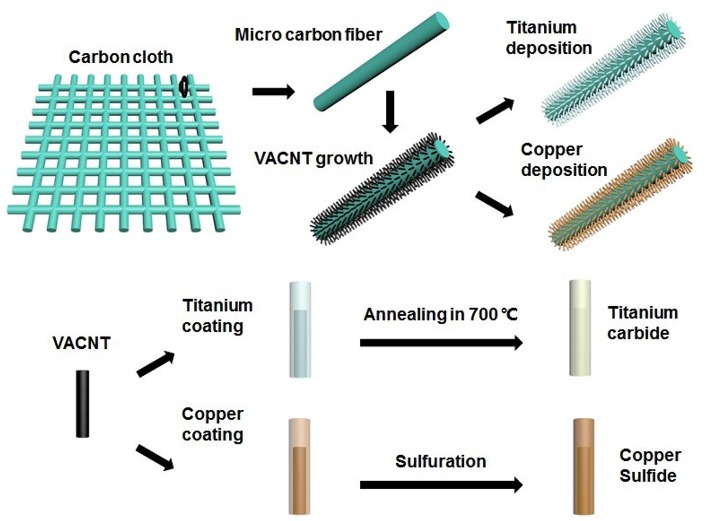
Schematics of the fabrication processes of the cathode and anode electrode.

The fabrication process of the TiC@VCC and Cu_x_S@VCC electrodes is shown in Figure 1. A 20 nm nickel thin film functioning as catalyst for VACNT growth was deposited on the carbon cloth substrate through an electron beam evaporation system. The VACNT array synthesis was conducted in a plasma-enhanced chemical vapor deposition system (Wang et al., 2014b) to form 3D nanostructured VCC electrodes.

**Figure 2:**
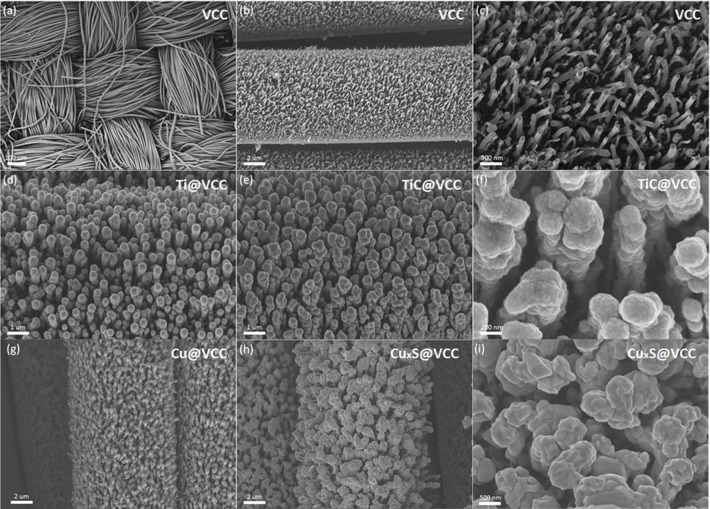
Morphology characterizations of VCC electrodes before and after the metal coating and thermal anneal functionalization. SEM images of VCC with different magnifications of 80 **(a)**, 5,000 **(b)** and 20,000 **(c)**. SEM images of Ti@VCC electrode before **(d)** and after **(e,f)** the functionalization. SEM images of Cu@VCC electrode before **(g)** and after **(h,i)** the functionalization.

SEM images of the VCC substrate in Figures 2a–c show that VACNTs were well distributed on the surface of the carbon cloth. The density and uniformity of the as-prepared VACNTs are much better than randomly grown carbon nanotube networks (De Volder et al., 2013) and common carbon nanotube arrays grown via thermal CVD system (Jiang et al., 2013). In addition, large interspacings in the VACNT array in Figure 2c can reserve space for later accommodation of active materials.

After the fabrication of VCC electrodes, titanium and copper were deposited on these VCC substrates via sputtering. SEM images of Ti@VCC (Figure 2d) and Cu@VCC (Figure 2g) electrodes suggest that the diameters of 1D nanostructures in both electrodes were uniformly increased, indicating that conformable coatings of titanium and copper on VCC electrodes have been achieved. Subsequently, high temperature annealing processes were conducted for Ti@VCC and Cu@VCC electrodes, as described in Section Preparation of TiC@VCC and CuxS@VCC electrodes. It is confirmed that the proposed *in-situ* functionalization process has successfully converted the two metal-coated VCC electrodes into TiC@VCC (Yildirim and Ciraci, 2005) and Cu_x_S@VCC (Vas-Umnuay et al., 2015) electrodes, respectively. In Figures 2e,h, it is seen that the diameters of metal coated VACNTs have not been significantly changed. From zoom-in images as seen in Figures 2f,j, the surface morphologies of 1D nanostructures in both electrodes have been altered, especially for the Cu_x_S@VCC electrode. It is also noticed that a notable aggregation occurred in the functionalized VACNT array during the annealing process, and part of the nanowires have stick together to form thicker bundles (Sun et al., 2015). Nevertheless, enough interspacing and porosity were reserved inside of these 3D nanostructured electrodes although the 1D nanostructured array would aggregate during the annealing step.

**Figure 3:**
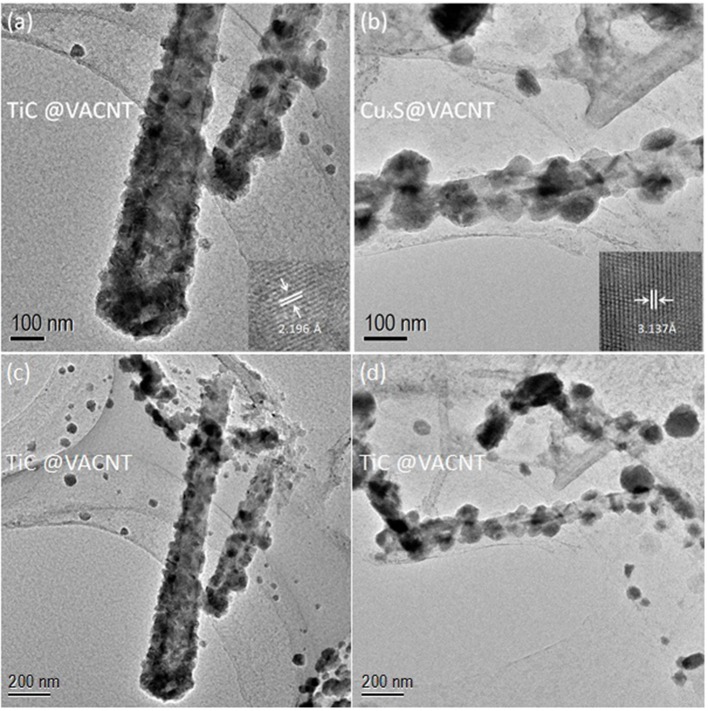
TEM images of electrodes: TiC@VACNT **(a,c)**, and Cu_x_S@VACNT **(b,d)**. (Insets: corresponding high resolution TEM images.

**Figure 4:**
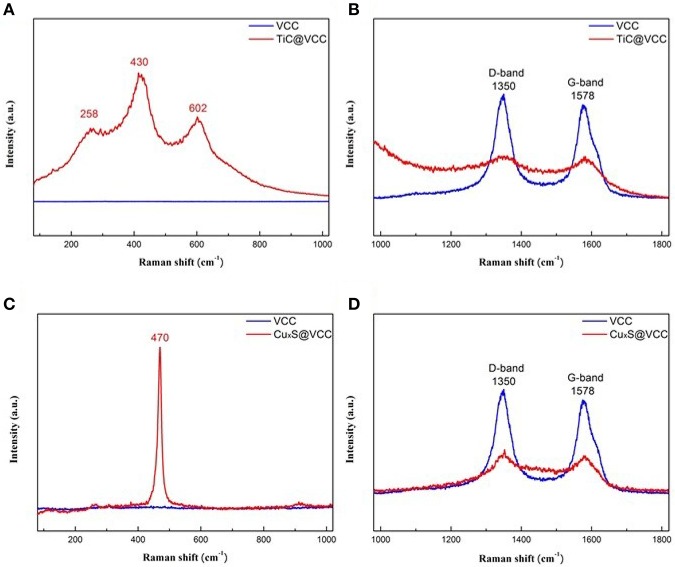
Raman spectra of TiC@VACNT electrode **(A,B)** and Cu_x_S@VACNT electrode **(C,D)**.

To obtain detailed nanostructures and materials compositions of the electrodes, transmission electron microscopy (TEM) characterizations were performed. Figures 3a,c reveil the core-shell nanostructure within a single TiC-coated VACNT. The inset of a high resolution TEM (HRTEM) image indicates a lattice interspacing of 2.196 Å, corresponding to the (002) planes of TiC (Xia et al., 2015). TEM image of a single Cu_x_S-coated VACNT (Figure 3d) is consistent with the SEM characterization results, and the aggregation phenomenon in Cu_x_S@VCC further escalated during the high-temperature functionalization. In this process, as-deposited Cu thin film was converted into Cu_x_S nanoparticles attached to the VACNTs with an approximate average diameter of 100 nm. The inset of Figure 3b shows a d-spacing of 3.127 Å, which coincides with other findings of high-temperature fabricated Cu_x_S (Quintana-Ramirez et al., 2014; Bulakhe et al., 2016). Low-magnification images of TiC@VCC and Cu_x_S@VCC are further provided as shown in Figures 4C,D to confirm the material of electrodes.

The Raman spectra of the TiC@VCC electrode and bare VCC electrodes are shown in Figures 4A,B, respectively. Strong characteristic peaks of 258, 430, and 620 cm^−1^ are attributed to TiC after functionalization (Lohse et al., 2005; Xia et al., 2015). Concurrently, the intensities of the Raman disordered band (D band) and graphitic band (G band) are significantly reduced, confirming that the TiC thin film shell was conformably coated on VACNTs. The Raman spectra of Cu_x_S@VCC electrodes illustrate a strong copper sulfide peak at 470 cm^−1^ (Figure 4C), and weakened D and G band (Figure 4D), which are well-consistent with other reported results (Munce et al., 2007; Quintana-Ramirez et al., 2014; Bulakhe et al., 2016).

**Figure 5:**
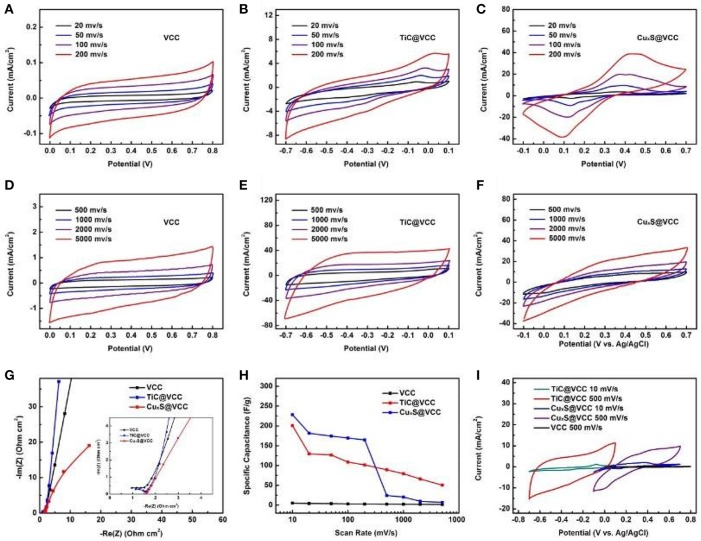
Electrochemical properties of the proposed electrodes. CV curves of the VCC **(A,D)**, TiC@VCC **(B,E)** and Cu_x_S@VCC **(C,F)** at different scan speeds from 10 to 5,000 mV/s. Electrochemical impedance **(G)**, Specific capacitance of electrodes **(H)** at different scan rates calculated from the CV curves **(A,F)**. Comparative CV curves of VCC, TiC@VCC and Cu_x_S@VCC electrodes performed in a three-electrode cell **(I)**.

The electrochemical characterizations of the proposed electrodes were performed in a standard three-electrode testing system with Pt plate as a counter electrode, and Ag/AgCl as a reference electrode. TiC@VCC anode/Cu_x_S@VCC cathode were used as the working electrodes, and LiCl as an electrolyte. For comparison purpose, the CV curves of VCC electrodes at different scan speeds from 20 to 5,000 mV s^−1^ are first shown in Figures 5A,D. Correspondingly, the CV curves of the TiC@VCC anode (from −0.7–0.1 V, vs. Ag/AgCl) and Cu_x_S@VCC cathode (from −0.1–0.7 V, vs. Ag/AgCl) are presented in Figures 5B,E,C,F, respectively. It is confirmed that both TiC@VCC and Cu_x_S@VCC electrodes exhibit good capacitive behaviors at these scan speeds. Figure 5G further shows the real and imaginary parts of the electrochemical impedance for both electrodes, validating highly conductive properties with small impedance. The specific capacitance of TiC@VCC and Cu_x_S@VCC electrodes can be calculated from these CV curves, yielding much higher values in comparison with VCC electrodes for a scan rate up to 200 mV/s (Figure 5H). It is noticed that the specific capacitance of the CuxS electrode decreases sharply after 200 mV/s, which may limit the applications of the proposed electrode at lower scan rate. This is attributed to the limited migration of electrolyte ions at higher scan rates, and some similar results have been observed in other metal sulfide based electrodes (Choudhary et al., 2015). Nevertheless, the specific capacitance is still higher than carbon only electrodes at higher scan rates. Measured CV curves of TiC@VCC and Cu_x_S@VCC electrodes in the range of −0.7–0.7 V and scan speeds at 10 and 500 mV/s (Figure 5I) show a better rectangular shape than those of the VCC electrodes, suggesting dominating capacitive characteristics.

**Figure 6:**
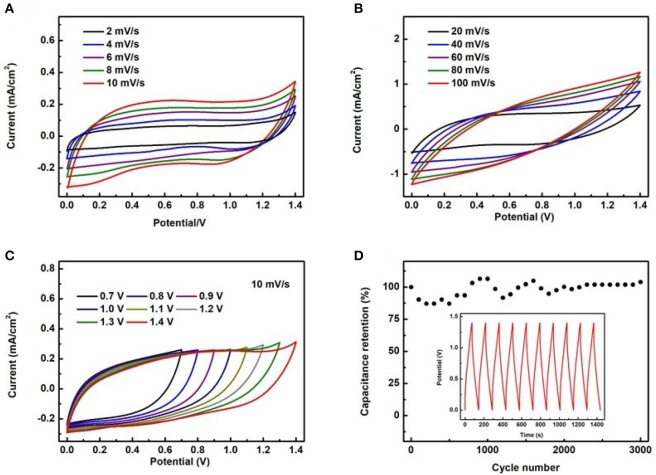
The electrochemical properties of the full device. CV curves of the full device at different scan rates **(A,B)**, CV curves at different bias voltages under a scan rate of 10 mV/s **(C)**, capacitance retention vs. cycle number for the full device **(D)**.

To evaluate the applicability of the proposed electrodes, a full device was assembled using the TiC@VCC as anode and the Cu_x_S@VCC as cathode, having a working range from 0 to 1.4 V. Figures 6A,B illustrate the measured CV curves of the full device at scan rates from 2 to 100 mV/s. At these scan speeds, good rectangular shapes and large curve areas are obtained. The CV curves as a function of the bias voltage (from 0.7 to 1.4 V) at a scan rate of 10 mV/s (Figure 6C) confirm that the full device can operate at different bias voltages, and therefore, can operate effectively as a micro-supercapacitor. After the charging and discharging test of 3,000 cycles, it is found that the specific capacitance retention is from ~80% to 110% of its original value (Figure 6D), which is relatively unstable when compared with other demonstrated micro-supercapacitors. This phenomenon is attributed to the side electrochemical reactions occurred during the first several hundred cycles within the copper sulfide in positive electrode (Zhu et al., 2012; Hsu et al., 2014; Bulakhe et al., 2016). Experimental results of copper sulfide based supercapacitors suggest that the unstable cycling performance in full device was a common issue and usually only 1,000~2,000 stable cycles of electrochemical test can be achieved. Nevertheless, all these results suggest that the full device shows an excellent electrochemical performance when compared to other recently reported full micro-supercapacitors.

## Conclusions

In this paper, we designed, fabricated and experimentally demonstrated a metal-based asymmetric supercapacitor using TiC@VCC as anode and Cu_x_S@VCC as cathode. TiC and Cu_x_S were prepared through *in-situ* functionalizations on the VCC substrate, providing a large interspacing and porosity to the 3D nanostructured electrodes. The electrodes showed a specific capacitance of 200.89 and 228.37 F g^−1^ in the potential window of −0.7 to 0.1 V and −0.1 to 0.7V, respectively. A full device assembled from the electrodes was able to work within a potential window of 0–1.4 V at a scan speed up to 100 mV/s, and demonstrated a maximum energy density of 9.12 Wh kg^−1^ at a power density of 46.88 W kg^−1^. Cycling measurements showed that the capacitance retention was between 80 and 110% of its original value. The proposed all-solid-state asymmetric supercapacitor demonstrated a high applicability and can be used as efficient energy-storage devices.

## Data Availability

The datasets generated for this study are available on request to the corresponding author.

## Author Contributions

LS and QZ conceived the idea. LS, XW, and YuW designed and fabricated the sample, and conducted the experiment. All the authors contributed to the analysis of data and the draft of the manuscript.

### Conflict of Interest Statement

The authors declare that the research was conducted in the absence of any commercial or financial relationships that could be construed as a potential conflict of interest.
